# Comparison of paraspinal muscle degeneration and decompression effect between conventional open and minimal invasive approaches for posterior lumbar spine surgery

**DOI:** 10.1038/s41598-020-71515-8

**Published:** 2020-09-03

**Authors:** Chen-Ju Fu, Wen-Chien Chen, Meng-Ling Lu, Chih-Hsiu Cheng, Chi-Chien Niu

**Affiliations:** 1 Bone and Joint Research Center, Chang Gung Memorial Hospital, Linkou, Taoyuan Taiwan, ROC; 2 Division of Emergency and Critical Care Radiology, Department of Medical Imaging and Intervention, Chang Gung Memorial Hospital, Linkou, Taiwan, ROC; 3 Department of Orthopedic Surgery, Chang Gung Memorial Hospital, Taoyuan, Taiwan, ROC; 4 Department of Orthopedic Surgery, Chang Gung Memorial Hospital, Kaohsiung, Taiwan, ROC; 5 College of Medicine, Chang Gung University, Taoyuan, Taiwan, ROC; 6 School of Physical Therapy and Graduate Institute of Rehabilitation Science, College of Medicine, Chang Gung University, Taoyuan, Taiwan, ROC; 7 Department of Orthopaedic Surgery, Chang Gung Memorial Hospital, Linkou, Taiwan, ROC

**Keywords:** Outcomes research, Medical research

## Abstract

Laminotomy and transforaminal lumbar interbody fusion (TLIF) is usually used to treat unstable spinal stenosis. Minimally invasive surgery (MIS) can cause less muscle injury than conventional open surgery (COS). The purpose of this study was to compare the degree of postoperative fatty degeneration in the paraspinal muscles and the spinal decompression between COS and MIS based on MRI. Forty-six patients received laminotomy and TLIF (21 COS, 25 MIS) from February 2016 to January 2017 were included in this study. Lumbar MRI was performed within 3 months before surgery and 1 year after surgery to compare muscle-fat-index (MFI) change of the paraspinal muscles and the dural sac cross-sectional area (DSCAS) change. The average MFI change at L2–S1 erector spinae muscle was significantly greater in the COS group (27.37 ± 21.37% vs. 14.13 ± 19.19%, P = 0.044). A significant MFI change difference between the COS and MIS group was also found in the erector spinae muscle at the caudal adjacent level (54.47 ± 37.95% vs. 23.60 ± 31.59%, P = 0.016). DSCSA improvement was significantly greater in the COS group (128.15 ± 39.83 mm^2^ vs. 78.15 ± 38.5 mm^2^, P = 0.0005). COS is associated with more prominent fatty degeneration of the paraspinal muscles. Statically significant post-operative MFI change was only noted in erector spinae muscle at caudal adjacent level and L2–S1 mean global level. COS produces a greater area of decompression on follow up MRI than MIS with no statistical significance on clinical grounds.

## Introduction

Lumbar spinal fusion with conventional open surgery (COS) is an effective method to relieve the symptoms of spinal stenosis and instability. However, COS is associated with extensive iatrogenic paraspinal muscle injury due to direct muscle dissection, denervation, and ischemia during long hours of muscle retraction. The paraspinal muscles, especially the multifidus and erector spinae muscles, are essential for carrying physiological loads and functional movement, and play an important role in the stability of the lumbar spine^1^. Muscle atrophy after COS occurs frequently, and can result in back pain and failed back surgery syndrome^2^. Therefore, minimally invasive surgery (MIS) techniques for lumbar spine fusion have become popular because they avoid long incisions and reduce muscle injury^1,3,4^. However, few studies have examined the differences of postoperative paraspinal muscle changes and outcomes between COS and MIS.

The purpose of this study was to compare the degree of postoperative fatty infiltration in paraspinal muscles and the degree of spinal decompression between COS and MIS based on magnetic resonance imaging (MRI).

## Materials and methods

### Patients and data collection

This study was approved by Chang Gung Memorial Hospital’s institution review board and all methods were performed in accordance with the relevant guidelines (approval no. 201702031B0). A written informed consent was obtained from every participant. All procedures in this study were performed in accordance with the relevant guidelines and regulations. This study adheres to STROBE statement guidelines (Supplementary Appendix 1).

This was a prospective, non-randomized, cohort study. Patients who underwent 1- or 2-level transforaminal lumbar interbody fusion (TLIF) by COS or MIS at our hospital from February 2016 to January 2017 were enrolled in the study.

Preoperative clinical symptoms included low back pain, sciatica, and variable neurological symptoms. The pathologies for surgery were segmental instability, spondylolisthesis, and disc degeneration disease with herniation and/or spinal stenosis. Patients with previous spine surgery, spine trauma, infection, ankylosing spondylitis, cancer, and congenital spinal deformities were excluded.

The functional scores (visual analogue scale [VAS] pain score, Oswestry Disability index [ODI], and Japanese Orthopedic Association [JOA] score) are collected pre-operation and 1 year post-operation. The plain radiographs (AP, lateral and flexion–extension views) was done 1 year after the surgery. The complete fusion was determined to be achieved if visible interbody osseous bridge had formed around the cage, no radiolucent line between the cage and endplate, and no motion on the dynamic flexion extension radiographs.

Lumbar MRI studies were obtained within 3 months before surgery and 1 year after surgery.

### Surgical technique

All patients received laminotomy, TLIF with cage insertion, and fixation with a transpedicle screw fixation device. Bilateral posterolateral fusion (inter-transverse fusion) was performed in addition to TLIF in the COS group. In the COS group, the paraspinal muscles were dissected away from the posterior elements (spinal process, lamina, and facet joints) via a midline approach, and laminotomy was performed at the index level with preservation of the adjacent supra- and inter-spinous ligament. In the MIS group, a fluoroscope-assisted percutaneous instrumentation technique via a para-midline approach and bilateral laminotomy by unilateral medial facetectomy for decompression were performed.

### MRI protocol

To avoid strong susceptibility artifacts around the postoperative metallic instruments, the MRI examinations were performed with a 1.5-T MR scanner (GE, Optima MR 450 W). The MR sequences included axial T1-weighted spin-echo (TR range/TE range 400–550/11–12) and axial T2-weighted spin-echo (TR range/TE range 4,000–4,300/95–120) sequences with a matrix size of 288 × 288, field of view of 200 × 170 mm, slice thickness of 4 mm, and interslice gap of 1 mm in the axial planes. The axial images were obtained at intervertebral disc levels horizontal to the endplate of the vertebral bodies from L2–3 to L5–S1. MR images were evaluated on a picture archiving and communication system work station (Centricity PACS, Radiology RA1000 Workstation, GE Healthcare).

**Figure 1:**
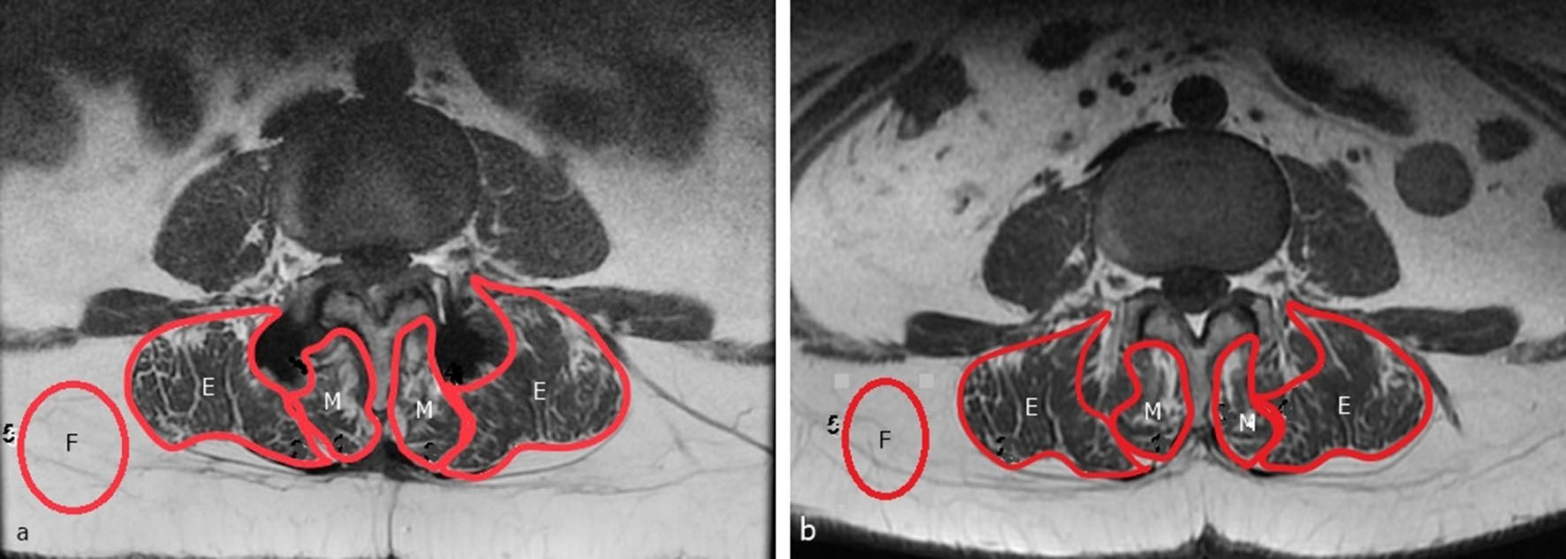
Illustration of the MFI evaluation technique. The mean SI in the ROI of the multifidus muscles (M), erector spinae muscle (E), and subcutaneous fat (F) were obtained from postoperative (**a**) and preoperative (**b**) T1WI MRI images using the picture archiving and communication system software tools. *MFI* mean SI of the target muscle/mean SI of subcutaneous fat.

The replacement of lean muscle by a fatty component suggests a decreased contractile muscle component and fatty degeneration of the muscle. The degree of fatty degeneration of the paraspinal muscle was determined by measuring the deposition of fat in the muscle. A muscle-fat-index (MFI) was used to reflect the amount of fatty infiltration in muscle using T1-weighted axial images. The MFI was defined as the mean signal intensity of the target muscle/mean signal intensity of homogenous subcutaneous fat^5,6^. The regions of interest (ROI) of individual muscles (multifidus muscle and rector spinae muscle) were measured on axial T1-weighted images by placing polygon points around the outer margins of the muscles to avoid metallic artifacts at each disc level from L2–3 to L5–S1, first on postoperative images and then on corresponding preoperative images (Fig. 1). The ROI of homogenous fat tissue was obtained from the homogenous subcutaneous tissue of the back in the same image. The mean signal intensity (SI) in the ROI of the target muscle and fat was obtained for calculating the MFI. The postoperative MFI change = (postoperative MFI − preoperative MFI)/preoperative MFI.

MFI was measured at the operated level, the cranial adjacent level, the caudal adjacent level, and from L2–3 to L5–S1. The global lumbar spine value was defined as the average of the values obtained from L2–3 to L5–S1.

**Figure 2:**
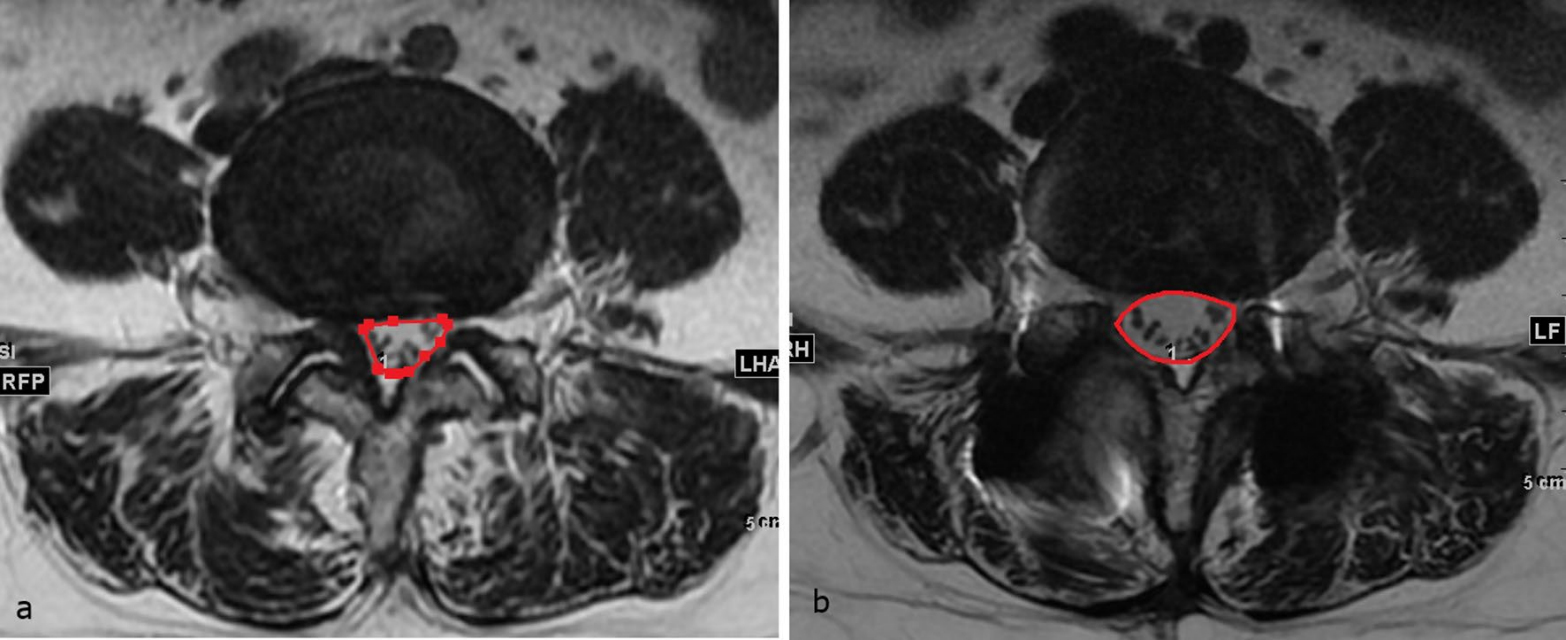
Illustration of the DSCSA evaluation technique. Preoperative MRI (**a**) showed dural sac compression due to a bulging disc and ligamentum flavum hypertrophy. The postoperative MRI (**b**) showed improvement of DSCSA after laminectomy.

The dural sac cross-sectional area (DSCSA) was measured at the most stenotic level on preoperative MR images, and at the same level on postoperative MRI T2-weighted axial images by polygon point measurement (Fig. 2). The improvement of spinal stenosis was defined as DSCSA change from before to after surgery.

All measurements were performed independently by 2 different observers who were blinded to the operative method. The average values of the 2 observations at each lumbar level were used for the analysis.

### Statistical methods

Comparison of postoperative MFI changes of the multifidus muscle and rector spinae muscle between the COS and MIS groups were performed using the unpaired *t* test. All statistical comparisons were 2-tailed, and the threshold for statistical significance was set at P < 0.05.

The inter-observer reliability was examined using 1-way analysis of variance, and the intra-class correlation coefficient (ICC).

## Results

**Figure 3:**
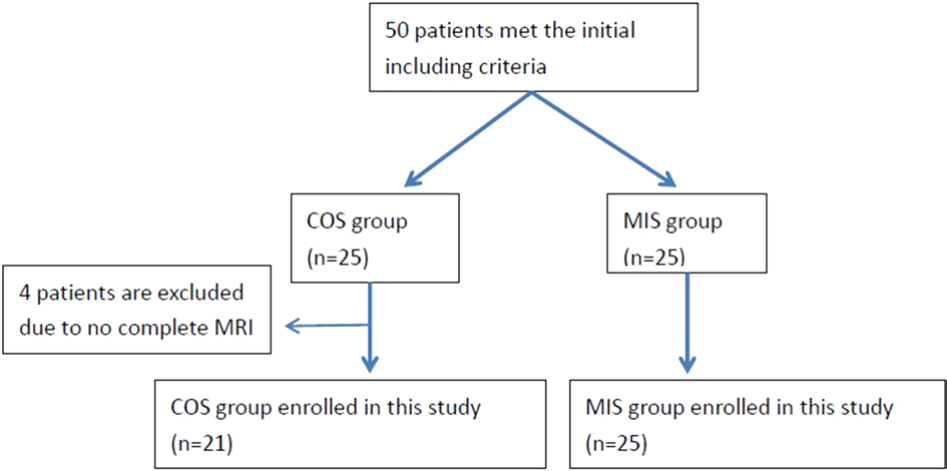
Flowchart of patient inclusion.

Initially there were 50 patients enrolled in this study. But 4 patients were excluded due to they did not complete the follow up MRI (2 patient suffered from other medial disease and 2 lost of follow up) (Fig. 3). Finally, total 46 patients (21 in COS group, 25 in MIS group) were included in this study. The mean age of patients in the COS group was 59.2 ± 8.6 years (range 41–77 years), and in the MIS group was 60.2 ± 10.0 years (range 38–78 years). Postoperative lumbar MRI studies were obtained a mean of 12.5 months after surgery in the COS group and 14.7 months after surgery in the MIS group. Patient demographic data, functional scores (visual analogue scale [VAS] pain score, Oswestry Disability index [ODI], and Japanese Orthopedic Association [JOA] score) and interbody fusion rate are summarized in Table 1. There was no significant difference in sex, age, functional scores, and interbody fusion rate between two groups.

**Table 1:** Patient demographic data.

Parameter	COS (n = 21)	MIS (n = 25)	P-value
Age (years)	59.2 ± 8.6	60.2 ± 10.0	0.757
Sex (F/M)	14/7	13/12	0.325
BMI (kg/m^2^)	26.9 ± 3.6	25.4 ± 2.4	0.152
Operated level			0.847
L2–3	0	1	
L3–4	0	2	
L4–5	15	16	
L3–5	3	1	
L5–S1	1	4	
L4–S1	2	1	
VAS (back)			
Pre-op	3.8 ± 2.7	3.2 ± 2.7	1.000
Post-op 1Y	1.0 ± 1.4	0.9 ± 1.7	0.99
VAS (leg)			
Pre-op	5.0 ± 3.3	3.8 ± 3.4	0.305
Post-op 1Y	0.5 ± 1.4	0.5 ± 1.5	0.72
ODI (%)			
Pre-op	31.8 ± 16.2	22.4 ± 14.6	0.168
Post-op 1Y	5.8 ± 6.2	4.2 ± 6.0	0.55
JOA			
Pre-op	53.1 ± 6.2	56.3 ± 9.6	0.20
Post-op 1Y	64.5 ± 6.8	65.5 ± 6.9	0.66
Fusion rate 1Y	95.24%	96%	0.9

The assessment of inter-observer reliability showed good agreement for the MFI (ICC = 0.78), and excellent agreement for the DSCSA measurement (ICC = 0.91), indicating the measurements were reliable.

**Figure 4:**
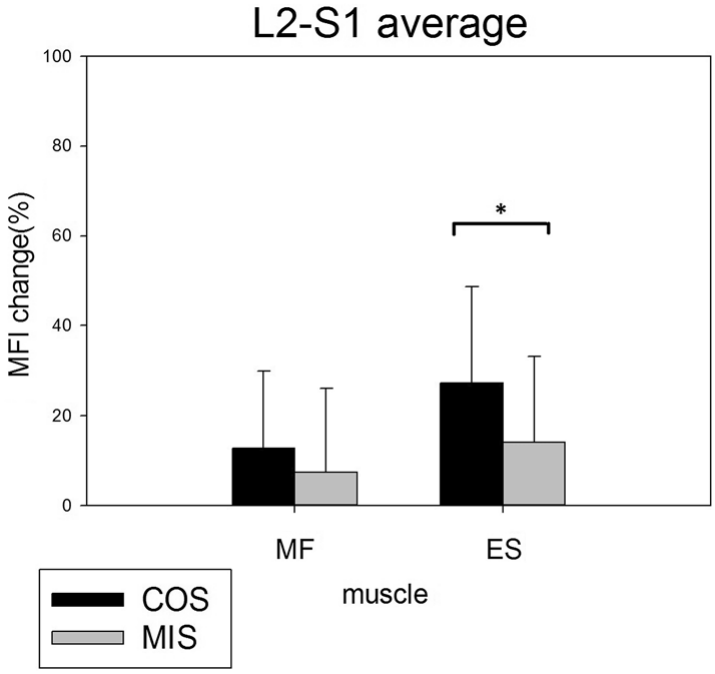
The mean global postoperative MFI change in the multifidus and erector spinae muscles from L2–S1. There was significantly increased fatty infiltration in the erector spinae muscle in the COS group.

The postoperative MFI was increased in both the multifidus and erector spinae muscles at all levels in both the COS and MIS groups on the 1-year postoperative MRI studies. The global MFI change of the multifidus muscle at L2–S1 was greater in the COS than the MIS group, but the difference was not statistically significant (12.85 ± 17.17% vs. 7.45 ± 18.76%, P = 0.343). The global MFI change of the erector spinae muscle at L2–S1 was significantly greater in the COS group (27.38 ± 21.37% vs. 14.14 ± 19.19%, P = 0.044) (Fig. 4).

**Figure 5:**
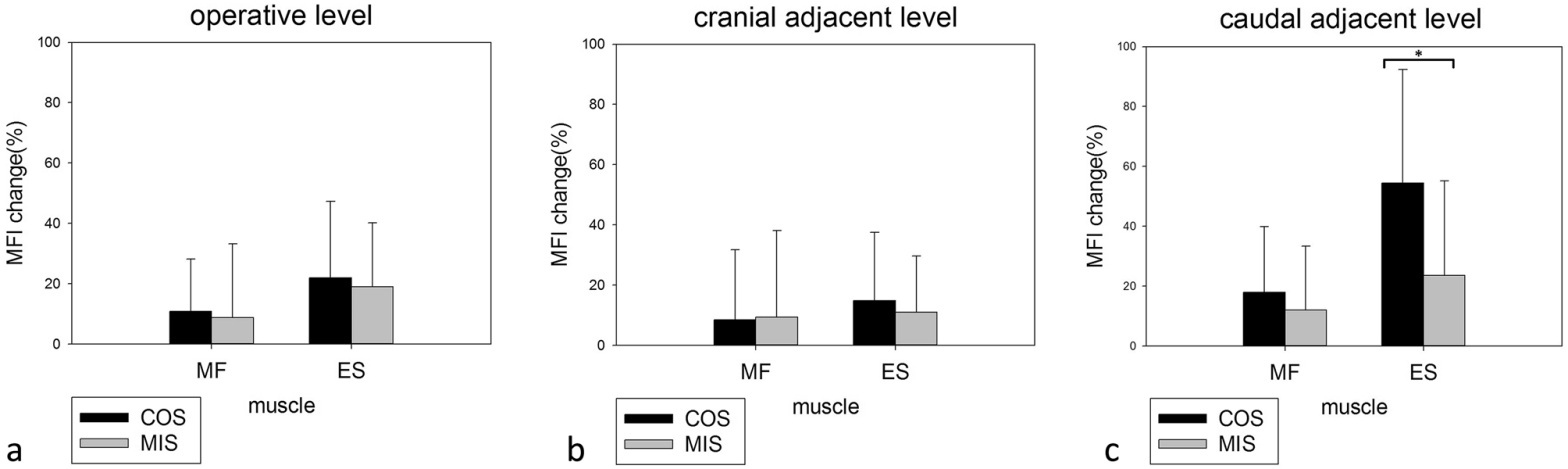
Postoperative MFI change in the multifidus and erector spinae muscles at the operated level (**a**), cranial adjacent level (**b**), and caudal adjacent level (**c**) at follow-up MRI 1 year after operation. There was a trend of more severe fatty infiltration in the COS group. A significant MFI change difference between the two methods was only noted in the erector spinae muscle at the caudal adjacent level.

The MFI changes at the operated and the cranial and caudal adjacent levels were compared between the two groups (Fig. 5). There was a greater trend of increasing fat infiltration after COS than MIS at all three levels of the paraspinal muscle, except for the multifidus muscle at the cranial adjacent level. However, the only significant MFI change difference between the COS and MIS groups was found at the caudal adjacent level of the erector spinae muscle (54.48 ± 37.95% vs. 23.61 ± 31.59%, P = 0.016).

**Figure 6:**
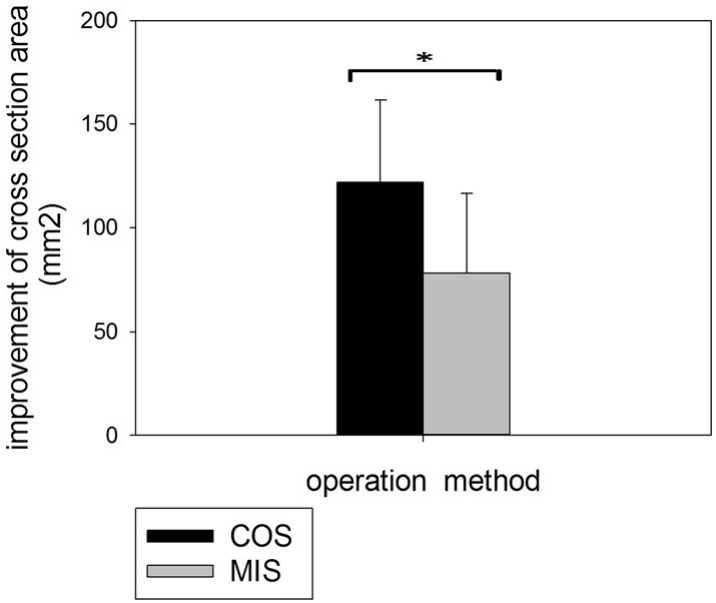
Improvement of DSCSA after operation was more prominent in the COS group than in MIS group (P = 0.0005).

The average improvement of DSCSA postoperatively was significantly greater in the COS group (128.15 ± 39.83 mm^2^) compared to the MIS group (78.15 ± 38.5 mm^2^) (P = 0.0005) (Fig. 6). All functional scores (VAS, ODI. and JOA) were improved 1 year postoperatively; however, there were no significant differences between the two groups (Table 1).

## Discussion

Posterior spinal fusion surgery is effective for treating degenerative spine pathologies with spinal stenosis and instability. In COS, the paraspinal muscles need to be deflected from the spinous processes, and this can cause muscle denervation, ischemic, and progressive muscle atrophy^2,7–9^. A number of MIS methods have been developed to avoid extensive muscle dissection, and have become popular.

Several studies have compared postoperative paraspinal muscle degeneration occurring after COS and MIS using MRI or computed tomography (CT) (Table 2)^1,3,9–15^. Mori et al. evaluated muscle damage from COS using the spinous process-splitting approach for decompression, and found significant postoperative multifidus muscle atrophy at the operated and caudal adjacent levels^15^. Min et al. reported less multifidus muscle degeneration following MIS than COS surgery by measuring the average cross-sectional area and fat infiltration of muscle at the L1–5 levels^1^. Stevens et al. reported less edema and atrophy of the multifidus muscle in patients that received MIS as compared to those that received COS at 6 months postoperatively^13^.

**Table 2:** Imaging studies comparing postoperative paraspinal muscle changes between conventional open and minimal invasive surgery.

Author	Surgical technique	Measurement method	Measurement muscle/level	Patient number	Mean follow-up time (months)	Change of paraspinal muscle
Min et al.^1^	TLIF/PSF	MRI-fat infiltration/GCSA	MF + ES/L1–5	48	12	Fat Open: 6.63% MIS: 2.14% GCSA Open: − 0.14% MIS: − 0.08%
Kim et al.^9^	PLIF/Laminectomy	MRI-GCSA	MF/adjacent level	19	21	Open: − 30.35% MIS: − 3.68%
Fan et al.^3^	PLIF/PSF	MRI-FCSA	MF/op + adjacent level	16	12	OP level Open: − 36.8%, MIS: − 12.2% Adjacent level Open: − 29.3%, MIS: − 8.5%
Bresnahan et al.^11^	Posterior decompression	MRI-FCSA	MF + ES/op level	18	16	Open: − 5.4% MEDS: 9.9%
Hyun et al.^14^	TLIF/PSF	CT-GCSA	MF/adjacent level	26	6	MA: − 20.7% PIA: − 4.8%
Mori et al.^15^	TLIF/PLIF/LPSF	MRI-GCSA atrophy radio	MF/op + adjacent level	53	36	OP level Open: 0.84, SPS: 0.96 Adjacent level Open: 0.80, SPS: 0.92
Tsutsumimoto et al.^16^	PLIF/PSF	MRI-GCSA atrophy radio	MF/adjacent level	20	12	Open: 0.74–0.62 MIS: 0.85–0.97
Stevens et al.^13^	PLF/PSF	MRI-T2 relaxion time/atrophy radio	MF/op level	8	6	T2 relaxion time Open: 99 ms, MIS: 51 ms Atrophy radio Open: 0.9%, MIS: 1.4%

*PSF* pedicle screw fixation, *GCSA* gross cross-sectional area, *FCSA* functional cross-sectional area, *PLIF* posterior lumbar interbody fusion, *TLIF* transforaminal lumbar interbody fusion, *MA* traditional midline approach, *PIA* paramedian interfascial approach, *SPS* spinous process-splitting, *MEDS* microendoscopic decompression of stenosis, *PLF* posterolateral lumbar fusion.

MRI provides a safe method for quantitative and qualitative evaluation of muscle without ionizing radiation. The only disadvantage of MRI for postoperative paraspinal muscle evaluation is artifacts due to the metallic implants. Most studies evaluating the paraspinal muscle postoperatively have used a cross-sectional area measurement. But we found that the metallic artifacts due to pedicle screws and rods on MRI result in poor inter-observer reliability of cross-sectional area measurement. For this reason, we used the MFI to reflect the degree of fatty infiltration in the paraspinal muscles^5,6^. Increased muscle fatty infiltration reflects decreased muscle quality, and might be a contributing factor of back pain^6^. Postoperative muscle edema and swelling can be present for up to 6 months after surgery, before it begins to subside^13,17^. For this reason, we performed the follow-up MRI evaluations at approximately 1 year after surgery, and used T1-weighted images for MFI qualification^18^.

The results of our study showed a trend of more severe postoperative fatty infiltration in both the multifidus and erector spinae muscle in the COS group than in the MIS group. In addition, the results showed muscular fatty degeneration was more prominent in the paraspinal muscles, consistent with similar studies in the literature. A significantly greater increase of MFI in the erector spinae muscle at caudal adjacent level was found in the COS group. While fatty infiltration of the erector spinae muscle at the operated level and the cranial adjacent level, and of the multifidus muscle at the operated, cranial, and caudal adjacent levels were all greater in the COS group, differences between the COS and MIS group were not significant.

Several studies have shown that fatty infiltration in the paraspinal muscles is more prominent in the lower lumbar segments in asymptomatic persons^18,19^. Olivier et al. found alteration of the contractile component of the cross-section area in the erector muscle mainly occurs distal to the lumbar operated segment^20^. In our study, fatty infiltration in postoperative muscle was also more prominent at the caudal adjacent level then the cranial adjacent level. This finding is likely because fatty infiltration of skeletal muscle usually begins at more distal skeletal locations^21^. As such, we would expect that postoperative muscular fatty atrophy will be earlier detected at lower lumbar levels.

Julio et al. suggested that single-level radiological morphology of the paraspinal muscle was not representative of the whole lumbar spine, and multi-level evaluation was more suitable for research of the entire lumbar musculature^22^. In this study, we evaluated global postoperative paraspinal muscular change by using the average MFI of L2–S1. We found increased postoperative fatty infiltration in the multifidus and erector spinae muscles in both groups, but a significant MFI change between the two operative methods was only found in the erector spinae muscle.

MIS laminectomy/laminotomy can provide good results of decompression of spinal stenosis as compared with COS^23,24^. To our knowledge, no study comparing postoperative DSCSA changes between COS and MIS has been published. DSCSA has been reported to be significantly associated with clinical outcomes after surgery for lumbar spinal canal stenosis^25^. Our evaluation of postoperative DSCSA changes indicated better decompression in the COS group than the MIS group. But all functional scores (VAS, ODI, and JOA) 1 year postoperatively were not different between the two groups. This finding suggests that the degree of decompression may not be the only factor that determines postoperative functional improvement.

There are some limitations to this study. For MFI evaluation, the preoperative ROI corresponding to the postoperative ROI was drawn manually by visualization. This may have resulted in some error; however, there was good inter-observer reliability. This was a single-center study with a small sample size, and only 1-year follow-up. Further studies with larger sample size and long-term follow-up are required to confirm the results.

## Conclusions

There was a trend of more severe fatty degeneration of the paraspinal muscles after COS than MIS. A statistical significant MFI difference between the two groups was only noted in the erector spinae muscle at the caudal adjacent level, and L2–5 mean global level. Postoperative muscular fatty infiltration was more prominent (may be more detectable) at the lower lumbar level. Decompression of spinal stenosis was significantly greater in the COS group as determined by DSCSA, but there was no significant difference of functional scores between the two groups at 1-year follow-up.

## Supplementary information

Supplementary Information.
