# Supplementary material for: Comparison of paraspinal muscle degeneration and decompression effect between conventional open and minimal invasive approaches for posterior lumbar spine surgery
Source: Sci Rep. 2020 Sep 3;10:14635. doi: 10.1038/s41598-020-71515-8 (PMC7471290; doi:10.1038/s41598-020-71515-8)
Supplement: Supplementary file 1 — Supplementary Information. [file 41598_2020_71515_MOESM1_ESM.docx]

**Appendix 1**

STROBE Statement—Checklist of items that should be included in reports of ***cohort studies***

|  | Item0No | Recommendation Page No |
| --- | --- | --- |
| **Title and abstract** | 1 | (*a*) Indicate the study’s design with a commonly used term in the title or the abstract 2 |
|  |  | (*b*) Provide in the abstract an informative and balanced summary of 2  what was done and what was found |
| Introduction | | |
| Background/rationale | 2 | Explain the scientific background and rationale for the investigation being reported 3 |
| Objectives | 3 | State specific objectives, including any prespecified hypotheses 3 |
| Methods | | |
| Study design | 4 | Present key elements of study design early in the paper 3-4 |
| Setting | 5 | Describe the setting, locations, and relevant dates, including periods of recruitment, exposure, follow-up, 3-4  and data collection |
| Participants | 6 | 1. Give the eligibility criteria, and the sources and methods of selection of participants. 3-4   Describe methods of follow-up |
|  |  | (*b*) For matched studies, give matching criteria and number of exposed and Not applicable  unexposed |
| Variables | 7 | Clearly define all outcomes, exposures, predictors, potential confounders, and effect modifiers. 4-6  Give diagnostic criteria, if applicable |
| Data sources/ measurement | 8 | For each variable of interest, give sources of data and details of methods of assessment 4-6  Describe comparability of assessment methods if there is more than one group |
| Bias | 9 | Describe any efforts to address potential sources of bias 6 |
| Study size | 10 | Explain how the study size was arrived at 3-4 |
| Quantitative variables | 11 | Explain how quantitative variables were handled in the analyses. If applicable, 6  describe which groupings were chosen and why |
| Statistical methods | 12 | (*a*) Describe all statistical methods, including those used to control for confounding 6 |
|  |  | (*b*) Describe any methods used to examine subgroups and interactions 6 |
|  |  | (*c*) Explain how missing data were addressed 6 |
|  |  | (*d*) If applicable, explain how loss to follow-up was addressed 6 |
|  |  | (*e*) Describe any sensitivity analyses Not applicable |
| Results | | |
| Participants | 13* | 1. Report numbers of individuals at each stage of study—eg numbers potentially eligible,examined for eligibility, 6-7   confirmed eligible, included in the study, completing follow-up, and analysed |
|  |  | (b) Give reasons for non-participation at each stage 6 |
|  |  | (c) Consider use of a flow diagram 17 |
| Descriptive data | 14* | (a) Give characteristics of study participants (eg demographic, clinical, social)  and information on exposures and potential confounders 15 |
|  |  | (b) Indicate number of participants with missing data for each variable of interest 6 |
|  |  | (c) Summarise follow-up time (eg, average and total amount) 6-7 |
| Outcome data | 15* | Report numbers of outcome events or summary measures over time 6-7 |
| Main results | 16 | 1. Give unadjusted estimates and, if applicable, confounder-adjusted estimates and their precision 7-8   (eg, 95% confidence interval). Make clear which confounders were adjusted for  and why they were included |
|  |  | (*b*) Report category boundaries when continuous variables were categorized Not applicable |
|  |  | (*c*) If relevant, consider translating estimates of relative risk into absolute risk Not applicable  for a meaningful time period |
| Other analyses | 17 | Report other analyses done—eg analyses of subgroups and interactions, Not applicable  and sensitivity analyses |
| Discussion | | |
| Key results | 18 | Summarise key results with reference to study objectives 8-10 |
| Limitations | 19 | Discuss limitations of the study, taking into account sources of potential bias or imprecision.  Discuss both direction and magnitude of any potential bias 10 |
| Interpretation | 20 | Give a cautious overall interpretation of results considering objectives, 8-10, 16  limitations, multiplicity of analyses, results from  similar studies, and other relevant evidence |
| Generalisability | 21 | Discuss the generalisability (external validity) of the study results 8-9 |
| Other information | | |
| Funding | 22 | Give the source of funding and the role of the funders for the present study and, 13  if applicable, for the original study on which the present article is based |
